# Dialectical Behavior Therapy as an intervention for Treatment Resistant Depression in adults: A protocol for systematic review and meta-analysis

**DOI:** 10.1371/journal.pone.0303967

**Published:** 2024-05-22

**Authors:** Larissa Junkes, Bruno R. Gherman, Jose Carlos Appolinario, Antonio E. Nardi

**Affiliations:** Institute of Psychiatry, Federal University of Rio de Janeiro, Rio de Janeiro, Brazil; University Hospital Cologne: Uniklinik Koln, GERMANY

## Abstract

**Background:**

Major Depressive Disorder is a long-term, recurring, and very common illness that is associated with a significant decline in functional ability. The gold-standard method of treating depression is pharmacotherapy, which involves the use of antidepressant medications either alone or in various combinations. However, approximately 30% of Major Depressive Disorder patients suffer from Treatment Resistant Depression, a more severe condition that has a profound impact on patients’ lives. Our study aims to conduct the first comprehensive review and meta-analysis to assess the effectiveness and safety of adding Dialectical Behavior Therapy to antidepressant medications compared to groups using pharmacotherapy alone as an intervention for adults with Treatment Resistant Depression.

**Materials and methods:**

We will search for publications in the following databases: Cochrane Central Register of Controlled Trials, MEDLINE, Embase, Lilacs, Web of Science, and PsycINFO. We will manually review the reference lists of the included studies to identify potentially relevant studies. There will be no restrictions on the language or publication date. Quality assessment of the included studies will be performed independently according to the Cochrane Risk of Bias instrument. To assess the certainty of the findings’ body of evidence, we will use the Grading of Recommendations Assessment, Development and Evaluation (GRADE) methodology. This study aims to determine the effectiveness and safety of Dialectical Behavior Therapy as an intervention for Treatment Resistant Depression in adults.

**Ethics and dissemination:**

Ethical approval was not required as individual patient data was not obtained. Our intention is to publish the systematic review in a medical journal that offers open access upon completion of the process.

**Trial registration:**

**PROSPERO registration number** CRD42023406301. Registered on March 24, 2023.

## Introduction

Depression is one of the most common and widely spread conditions in the population, impacting over 300 million people worldwide, and is linked to substantial functional impairment [1,2]. Patients with this condition, particularly the subgroup with Treatment Resistant Depression (TRD), present a greater challenge due to the severity of their symptoms, longer duration of illness, high rates of recurrence and comorbidities, and increased mortality [3,4]. According to the STAR*D study, TRD accounts for 30% of all Major Depressive Disorder (MDD) cases, with an estimated prevalence of 1% in the population [5]. So far, there is no single definition of TRD in the current medical literature [6]. Some authors consider it a lack of response when at least one attempt of treatment with an antidepressant at an adequate dose for at least six weeks does not show improvements [7]; however, TRD is also defined as a failure to achieve remission after trying two or more correct courses of antidepressants [8–12]. In this review, Treatment Resistant Depression will be defined as the lack of a response to more than one attempt of treatment with an antidepressant at a sufficient dose for a minimum of six weeks.

Treating resistant depression is still difficult, even with the range of medications available for this specific group. Antidepressant medications from various classes, used either on their own, in combination, or enhanced by other drugs, are the most commonly utilized strategies [13]. Due to the significant magnitude and severe impact of this condition, further optimized interventions are required. The combination of psychotherapy and medication demonstrated symptomatic improvement, reduced risk of relapse, and higher adherence to depression treatment in comparison to using antidepressant drugs alone [14].

Concerns about the costs of mental health treatment have been increasing, leading to a rise in economic evaluation research. Due to limited resources, it is necessary to prioritize cost-effectiveness and make strategic, clinical, preventive and person-centered decisions [15]. Cognitive Behavioral Therapy (CBT), one of the most thoroughly studied psychotherapeutic interventions for depression, has been frequently evaluated in economic analyses. A systematic review of 22 studies published between 2004 and 2012 on CBT for Major Depressive Disorder found that the majority of studies showed "acceptable incremental cost-effectiveness ratios" [16]. Therefore, psychotherapy is a possible choice to enhance the effectiveness of treatment.

A Cochrane Systematic Review [17] assessed the effectiveness of psychotherapy in adults with TRD, including studies evaluating Cognitive Behavioral Therapy (CBT), Interpersonal Therapy (IT), Brief Psychodynamic Therapy (BPT), and Dialectical Behavior Therapy (DBT). Six studies were evaluated, the majority of them were small (except for one CBT trial which was large), and all studies were at high risk of detection bias for the main outcome of self-reported depressive symptoms. The research findings suggest that a combination of psychotherapy and medication is just as effective as using medication alone. However, the results should be interpreted with caution as most of the studies included were small and had a risk of bias in detecting self-reported depressive symptoms.

Dialectical Behavior Therapy, which was developed by American psychologist Marsha Linehan, was originally designed to help women who exhibit suicidal and self-harming behaviors [18–22]. Using content from Zen philosophy, DBT relies on dialectics to reach synthesis from two opposing views: the balance between changing what is under control and accepting the facts and experiences that cannot be changed. Since its development, Dialectical Behavior Therapy has been tested and implemented as a treatment in various clinical settings, from a transdiagnostic viewpoint, with initial evidence supporting its effectiveness [23]. A subgroup of patients who have been extensively evaluated with DBT is adolescents. In a systematic review of clinical efficacy conducted in Canada on DBT in adolescents (DBT-A) for suicide prevention, the authors noted that all included studies reported some clinical efficacy of using DBT in reducing suicidality, including reductions in self-harming behaviors and suicidal ideation. However, no statistically significant differences were observed in completed suicides among participants treated with DBT-A [24]. In another systematic review, where selected studies evaluated 1,673 adolescents, DBT-A demonstrated small to moderate effects in reducing self-harm (g = -0.44; 95% CI -0.81 to -0.07) and suicidal ideation (g = -0.31, 95% CI -0.52 to -0.09) compared to the control group. These results suggest that DBT-A shows promise as a treatment for reducing both self-harm and suicidal ideation in adolescents [25].

The standard DBT, which includes individual therapy sessions, skills training groups, telephone coaching, and consulting groups for professionals, has been gradually adjusted to meet the needs of patients diagnosed with conditions other than suicidal, self-harming behaviors and borderline personality disorder. For Treatment Resistant Depression, an adaptation consisting of a 16-session DBT skills training protocol was performed, [26] which showed significantly greater improvements in depressive symptoms as measured using the Hamilton Rating Scale for Depression (HAM-D) and Beck Depression Inventory (BDI) compared to the control group, who remained on drug therapy and the waiting list for skills group. A study conducted in Boston, USA [27] evaluated a Dialectical Behavioral Therapy skills training group for patients with Treatment Resistant Depression. The study showed positive results from training skills aimed at helping individuals process emotions in order to reduce depressive symptoms.

Adults with resistant depressive disorders may display traits of overcontrol, such as heightened self-criticism, diminished autonomy, rigid internalized expectations, heightened control over spontaneous emotions, and a fear of making mistakes [28–30]. Having too much self-control has been linked to being socially isolated, having difficulty relating to others, and showing emotions that are not genuine [31]. These maladaptive responses can prolong depressive symptoms [29,30]. Radically Open Dialectical Behavioral Therapy (RODBT) emphasizes the role of emotional expressions in forming social bonds and promoting empathetic behaviors. Therefore, RODBT has some evidence for use in Treatment Resistant Depression [28,32].

In England, the first RODBT Randomized Controlled Trial involved 250 patients in two groups: one would receive RODBT plus treatment as usual (TAU) and the other one received TAU alone. TAU was defined as any treatment provided by the British public health system or private treatment [33]. The intervention, RODBT, comprised 29 weekly individual sessions lasting one hour and 27 weekly skills classes lasting 2.5 hours. The treatments did not show a statistically significant difference in the primary endpoint after 12 months. However, after seven months, participants randomized to RODBT had substantially, and significantly, reduced depressive symptoms, relative to TAU, by 5.40 points in the Hamilton Depression Rating Scale. However, at the 12^th^ and 18^th^ month follow up, the difference of 2.15 (12 months) and 1.69 (18 months) points on the HDRS in favor of RODBT was not significant.

One of the reasons for testing the combination of DBT with pharmacotherapy in Treatment Resistant Depression is the effectiveness of DBT for individuals struggling to manage and regulate their emotions. This is a common challenge faced by patients with depression, who frequently display suicidal tendencies and intense anxiety. In DBT, patients are required to commit to making positive changes and concentrate on their present and future, rather than dwelling on their past, which is typical in depressive rumination. A robust, evidence-based study is necessary to clarify the role of Dialectical Behavior Therapy as an additional treatment with pharmacotherapy for Treatment Resistant Depression. This review study aims to meet this requirement, contributing to expanding the knowledge acquired and outlining for the specific subgroup of patients with TDR treated with DBT. Currently, no systematic reviews have been conducted that address this specific question. Therefore, the results of this study could help policymakers and public health professionals make informed decisions.

## Research question

What is the effectiveness and safety of Dialectical Behavior Therapy as an intervention for Treatment Resistant Depression in adults, in addition to standard drug treatment?

## Objectives

To assess how patients with Treatment Resistant Depression respond when Dialectical Behavior Therapy is added to their regular drug treatment, considering the following parameters:

- Depressive symptoms according to the Hamilton Depression Assessment Scale (HAM-D) and Beck Depression Inventory (BDI)
- Quality of life according to the SF-12
- Clinical global impression according to the CGI.

## Materials and methods

The protocol for this review was registered on PROSPERO on March 24, 2023 (registration number PROSPERO 2023 CRD42023406301). This systematic review will adhere to the Preferred Reporting Items for Systematic Reviews and Meta-Analyses (PRISMA guidelines) [34]. We will search for publications in the following databases: Cochrane Central Register of Controlled Trials (CENTRAL), MEDLINE, Embase, Lilacs, Web of Science, and PsycINFO. The reference lists of the included studies will be reviewed manually to identify potentially relevant studies. There will be no restrictions on the language or publication date. The following sensitive search strategy will be used (MEDLINE): (("Depressive Disorder, Treatment-Resistant"[Mesh] OR (Depressive Disorder, Treatment Resistant) OR (Depressive Disorders, Treatment-Resistant) OR (Disorder*, Treatment-Resistant Depressive) OR (Treatment-Resistant Depressive Disorder*) OR (Refractory Depression*) OR (Depression*, Refractory) OR (Therapy-Resistant Depression*) OR (Depression*, Therapy-Resistant) OR (Therapy Resistant Depression) OR (Treatment Resistant Depression*) OR (Depression*, Treatment Resistant) OR (Resistant Depression*, Treatment)) AND ("Dialectical Behavior Therapy"[Mesh] OR (Behavior Therapy, Dialectical) OR (Dialectical Behavior Therapies))) AND ("Psychotherapy"[Mesh] OR Psychotherapies).

### Selection criteria

The chosen articles must meet the following criteria: (1) The study participants must be 18 years old or older and have a diagnosis of Treatment Resistant Depression (TRD); (2) The Dialectical Behavior Therapy (DBT) intervention must be compared with other active treatments (such as medication or other forms of psychotherapy) or inactive treatments (like medication and waiting list); (3) The efficacy, focusing on improvement in depression symptoms as the primary outcome, should be measured using established and validated instruments; (4) Patients must have a categorical diagnosis of Depression and Treatment Resistant Depression; (5) The study designs that will be considered include randomized or open clinical trials, observational studies, and case series. Case reports will be excluded; (6) In regards to grey literature, manual searches and review of reference lists will be conducted to identify additional sources for investigation; (7) If there are other interventions used in combination with DBT, they will be assessed separately; (8) Children and adolescents under the age of 18 will not be included in the study and (9) Incomplete study protocols will not be considered.

### Studies selection

Initially, two authors (LJ and BG) will review titles and abstracts independently using predetermined criteria to determine eligibility and remove any duplicates using Rayyan software. Subsequently, they will individually evaluate the full text based on the specified criteria for inclusion in the review. Any disagreements will be resolved through consensus or, if necessary, by consulting a third party (JCA). The reasons for exclusion will be documented in the report based on a full-text review, and the selection process will be thoroughly documented to create a complete PRISMA study selection flowchart (Moher, 2009). If there are multiple publications on the same study, data from the most comprehensive outcomes will be included.

### Data extraction

Two authors (LJ and BG) will independently extract data using a form developed specifically for this review. If necessary, the authors will be contacted to obtain additional relevant or missing information. The data that will be collected includes authors, publication year, sociodemographic aspects, study design, intervention details such as the components of DBT delivered (individual DBT therapy, skills group, phone coaching, consultation team), treatment integrity procedures (treatment adherence, therapist competence, treatment differentiation–whether treatments differ from each other along critical dimensions), and the number of sessions for each component. Also collecting data on the DBT skills taught, such as whether all modules are covered (emotion regulation, interpersonal effectiveness, mindfulness, distress tolerance) or just a subset. Furthermore, information on any other psychotherapy components included with DBT, as well as any additional psychotherapeutic treatments used in the control groups, and the instruments used to evaluate depression diagnosis, participant eligibility criteria, use of intention-to-treat analysis (ITT), outcome data (including how outcomes were measured and any adverse effects reported), detailed information on interventions, study duration, and outcomes (including the clinical definition and measurement instrument used), pharmacological treatment details, adverse effects, whether clinical outcomes assessment is blinded, and information on participants who dropped out of the study.

### Quality assessment

Two of the authors (LJ and BG) will independently evaluate the methodological quality of the studies included using the Cochrane Risk of Bias (RoB2) instrument. RoB2 is utilized for systematic reviews that involve randomized clinical trials and is divided into five domains with ’signaling questions’ that provide additional information relevant for assessing bias risk. Response options to these ’signaling questions’ include: "yes", "probably yes", "probably not", "no", "no information", and "not applicable". Clear answers of ’yes’ and ’no’ often indicate strong evidence. The ’not applicable’ option is only used for questions with a non-mandatory response. Throughout the instrument’s application, responses are input into an algorithm which determines bias risk for each domain: high, low, or presence of any concerns about bias. If there is a discrepancy in results between the two authors, a third author will be consulted to come to a consensus. The risk of bias assessment will focus on participant selection, outcome measurement, and confounding control. This includes evaluating randomization, deviation from intended intervention, missing data, outcome measurement, and reporting of outcomes. Both primary and secondary outcomes will be assessed. Each outcome will be judged for risk of bias, categorized as ’low, high, or some concern’. Incomplete outcome data will be considered low risk of bias if dropouts are balanced across groups and unrelated to the outcome. Outcome selection bias will be assessed by comparing reported outcomes to published results. Intention-to-treat analysis will be used, with the population consisting of randomized patients who attend at least one DBT session (individual or skills group) and are evaluated post-baseline.

### Data analysis

If there are similar studies and it is possible to group them, a meta-analysis will be conducted. Statistical analyses will be performed using R 4.3.3 software. To measure the treatment effect, dichotomous outcome data will be analyzed using risk ratio (RR) with 95% confidence interval (CI). Continuous outcome data will be expressed as the mean difference if the outcome is measured in the same manner across the studies. Standardized mean differences will be used to combine studies measuring the same outcome, but with different instruments, if possible. We will contact the original investigators and ask for missing data if necessary.

Heterogeneity will be evaluated by visual inspection of the forest plot, together with evaluation of the chi-square test or we will calculate I^2^. It will be viewed as low or no heterogeneity if I^2^ < 50% while I^2^ ≥ 50% will be viewed as significant heterogeneity. If there is no statistical heterogeneity (I^2^ < 50% and P > 0.1), the fixed-effects model will be used for meta-analysis. If there is statistical heterogeneity (I >50% and P<0.1), the random-effects model will be used for meta-analysis. Consequently, we will conduct a sensitivity or subgroup analysis to identify possible sources of heterogeneity, if there is enough information available in the included studies, with the aim of estimating the effect of the intervention in different subsets of participants, in order to raise hypotheses.

Subgroup analysis will be performed on the following variables: gender, whether the patient was exposed to DBT in group or individual therapy, whether the patient was exposed to standard DBT or an adaptation, intervention duration, and classes and dosages of antidepressants being utilized. Sensitivity analysis will also be conducted using meta-regression, which will evaluate how these variables impact the outcome of the meta-analysis using regression techniques. This analysis will specifically be used for continuous variables like intervention duration. Furthermore, we will analyze publication bias using a funnel plot. The asymmetry of the funnel plot will be assessed using Egger’s test.

It is expected that the combined therapy group of Dialectical Behavior Therapy along with pharmacotherapy will demonstrate a more effective emotional regulation process compared to the group only exposed to medication. This is because mindfulness is expected to enhance emotional differentiation capacity, lead to quicker emotional recovery, reduce negative response to stress, and overall enhance emotional management processes, ultimately enhancing psychological well-being.

To assess the certainty of the findings’ body of evidence, we will use the Grading of Recommendations Assessment, Development, and Evaluation (GRADE) methodology, which evaluates five pillars: risk of bias, imprecision, inconsistency, indirect assessment of outcomes, and publication bias [35].

### Ethics and dissemination

Since this is a systematic review and meta-analysis, ethical approval is not required. The results of this review will be published in a journal that undergoes peer review and will also be presented at relevant conferences.

## Discussion

The seriousness of the consequences of depression, along with its continued high prevalence over the years, underscores the need for a thorough and up-to-date study of treatment options. There has been significant growth in pharmacological treatments, offering new ways to lessen the debilitating effects of the disease. Improved interventions are crucial in addressing this issue. Adding psychotherapy to drug treatment has the potential to yield positive results. However, there is limited evidence on which psychotherapeutic techniques are more effective for patients with Treatment Resistant Depression. The expected results may reveal that the efficacy of Dialectical Behavior Therapy combined with antidepressant medication in Treatment Resistant Depression is more enhanced than pharmacotherapy alone.

This planned review and meta-analysis will thoroughly examine the available evidence for TRD and the role of DBT in this specific subgroup. By collecting and summarizing information, this study aims to enhance our understanding of existing gaps and identify the most promising approaches to improve patient response. Furthermore, the findings of this review will guide future research efforts and inform healthcare practitioners.

## Supporting information

S1 Checklist PRISMA-P (Preferred Reporting Items for Systematic review and Meta-Analysis Protocols) 2015 checklist: Recommended items to address in a systematic review protocol*. (DOC)

S1 Dataset Draft of search strategy to be used with PubMed electronic database. (DOCX)
