# Supplementary material for: Dialectical Behavior Therapy as an intervention for Treatment Resistant Depression in adults: A protocol for systematic review and meta-analysis
Source: PLoS One. 2024 May 22;19(5):e0303967. doi: 10.1371/journal.pone.0303967 (PMC11111054; doi:10.1371/journal.pone.0303967)
Supplement: S1 Dataset — (DOCX) [file pone.0303967.s002.docx]

**S2 File. Draft of search strategy to be used with PubMed electronic database**

| **Components** | **Search items** | **Results** |
| --- | --- | --- |
| **#1** | **Treatment Resistant Depression:** "depressive disorder, treatment resistant"[MeSH Terms] OR ("depressive disorder, treatment resistant"[MeSH Terms] OR ("depressive"[All Fields] AND "disorder"[All Fields] AND "Treatment-Resistant"[All Fields]) OR "treatment-resistant depressive disorder"[All Fields] OR ("depressive"[All Fields] AND "disorder"[All Fields] AND "treatment"[All Fields] AND "resistant"[All Fields]) OR "depressive disorder treatment resistant"[All Fields]) OR ("depressive disorder, treatment resistant"[MeSH Terms] OR ("depressive"[All Fields] AND "disorder"[All Fields] AND "Treatment-Resistant"[All Fields]) OR "treatment-resistant depressive disorder"[All Fields] OR ("depressive"[All Fields] AND "disorders"[All Fields] AND "treatment"[All Fields] AND "resistant"[All Fields]) OR "depressive disorders treatment resistant"[All Fields]) OR ("disorder"[All Fields] AND "Treatment-Resistant"[All Fields] AND ("depressed"[All Fields] OR "depression"[MeSH Terms] OR "depression"[All Fields] OR "depressions"[All Fields] OR "depression s"[All Fields] OR "depressive disorder"[MeSH Terms] OR ("depressive"[All Fields] AND "disorder"[All Fields]) OR "depressive disorder"[All Fields] OR "depressivity"[All Fields] OR "depressive"[All Fields] OR "depressively"[All Fields] OR "depressiveness"[All Fields] OR "depressives"[All Fields])) OR ("Treatment-Resistant"[All Fields] AND ("depressed"[All Fields] OR "depression"[MeSH Terms] OR "depression"[All Fields] OR "depressions"[All Fields] OR "depression s"[All Fields] OR "depressive disorder"[MeSH Terms] OR ("depressive"[All Fields] AND "disorder"[All Fields]) OR "depressive disorder"[All Fields] OR "depressivity"[All Fields] OR "depressive"[All Fields] OR "depressively"[All Fields] OR "depressiveness"[All Fields] OR "depressives"[All Fields]) AND "disorder*"[All Fields]) OR (("refractories"[All Fields] OR "refractoriness"[All Fields] OR "refractory"[All Fields]) AND "depression*"[All Fields]) OR ("depression"[All Fields] AND ("refractories"[All Fields] OR "refractoriness"[All Fields] OR "refractory"[All Fields])) OR ("Therapy-Resistant"[All Fields] AND "depression*"[All Fields]) OR ("depression"[All Fields] AND "Therapy-Resistant"[All Fields]) OR ("depressive disorder, treatment resistant"[MeSH Terms] OR ("depressive"[All Fields] AND "disorder"[All Fields] AND "Treatment-Resistant"[All Fields]) OR "treatment-resistant depressive disorder"[All Fields] OR ("therapy"[All Fields] AND "resistant"[All Fields] AND "depression"[All Fields]) OR "therapy resistant depression"[All Fields]) OR (("therapeutics"[MeSH Terms] OR "therapeutics"[All Fields] OR "treatments"[All Fields] OR "therapy"[MeSH Subheading] OR "therapy"[All Fields] OR "treatment"[All Fields] OR "treatment s"[All Fields]) AND ("resist"[All Fields] OR "resistance"[All Fields] OR "resistances"[All Fields] OR "resistant"[All Fields] OR "resistants"[All Fields] OR "resisted"[All Fields] OR "resistence"[All Fields] OR "resistences"[All Fields] OR "resistent"[All Fields] OR "resistibility"[All Fields] OR "resisting"[All Fields] OR "resistive"[All Fields] OR "resistively"[All Fields] OR "resistivities"[All Fields] OR "resistivity"[All Fields] OR "resists"[All Fields]) AND "depression*"[All Fields]) OR ("depression"[All Fields] AND ("therapeutics"[MeSH Terms] OR "therapeutics"[All Fields] OR "treatments"[All Fields] OR "therapy"[MeSH Subheading] OR "therapy"[All Fields] OR "treatment"[All Fields] OR "treatment s"[All Fields]) AND ("resist"[All Fields] OR "resistance"[All Fields] OR "resistances"[All Fields] OR "resistant"[All Fields] OR "resistants"[All Fields] OR "resisted"[All Fields] OR "resistence"[All Fields] OR "resistences"[All Fields] OR "resistent"[All Fields] OR "resistibility"[All Fields] OR "resisting"[All Fields] OR "resistive"[All Fields] OR "resistively"[All Fields] OR "resistivities"[All Fields] OR "resistivity"[All Fields] OR "resists"[All Fields])) OR (("resist"[All Fields] OR "resistance"[All Fields] OR "resistances"[All Fields] OR "resistant"[All Fields] OR "resistants"[All Fields] OR "resisted"[All Fields] OR "resistence"[All Fields] OR "resistences"[All Fields] OR "resistent"[All Fields] OR "resistibility"[All Fields] OR "resisting"[All Fields] OR "resistive"[All Fields] OR "resistively"[All Fields] OR "resistivities"[All Fields] OR "resistivity"[All Fields] OR "resists"[All Fields]) AND "depression"[All Fields] AND ("therapeutics"[MeSH Terms] OR "therapeutics"[All Fields] OR "treatments"[All Fields] OR "therapy"[MeSH Subheading] OR "therapy"[All Fields] OR "treatment"[All Fields] OR "treatment s"[All Fields])) | 15,819 |
| **#2** | **Major Depressive Disorder:** "depressive disorder, major"[MeSH Terms] OR ("depressive disorder, major"[MeSH Terms] OR ("depressive"[All Fields] AND "disorder"[All Fields] AND "major"[All Fields]) OR "major depressive disorder"[All Fields] OR ("depressive"[All Fields] AND "disorders"[All Fields] AND "major"[All Fields]) OR "depressive disorders major"[All Fields]) OR (("major"[All Fields] OR "majored"[All Fields] OR "majoring"[All Fields] OR "majorities"[All Fields] OR "majority"[All Fields] OR "majors"[All Fields]) AND ("depressed"[All Fields] OR "depression"[MeSH Terms] OR "depression"[All Fields] OR "depressions"[All Fields] OR "depression s"[All Fields] OR "depressive disorder"[MeSH Terms] OR ("depressive"[All Fields] AND "disorder"[All Fields]) OR "depressive disorder"[All Fields] OR "depressivity"[All Fields] OR "depressive"[All Fields] OR "depressively"[All Fields] OR "depressiveness"[All Fields] OR "depressives"[All Fields]) AND "disorder*"[All Fields]) OR ("depressive disorder, major"[MeSH Terms] OR ("depressive"[All Fields] AND "disorder"[All Fields] AND "major"[All Fields]) OR "major depressive disorder"[All Fields] OR ("paraphrenia"[All Fields] AND "Involutional"[All Fields])) OR ("Involutional"[All Fields] AND "paraphrenia*"[All Fields]) OR ("depressive disorder, major"[MeSH Terms] OR ("depressive"[All Fields] AND "disorder"[All Fields] AND "major"[All Fields]) OR "major depressive disorder"[All Fields] OR ("paraphrenias"[All Fields] AND "Involutional"[All Fields])) OR ("depressive disorder, major"[MeSH Terms] OR ("depressive"[All Fields] AND "disorder"[All Fields] AND "major"[All Fields]) OR "major depressive disorder"[All Fields] OR ("psychosis"[All Fields] AND "Involutional"[All Fields]) OR "psychosis involutional"[All Fields]) OR ("depressive disorder, major"[MeSH Terms] OR ("depressive"[All Fields] AND "disorder"[All Fields] AND "major"[All Fields]) OR "major depressive disorder"[All Fields] OR ("Involutional"[All Fields] AND "psychoses"[All Fields]) OR "involutional psychoses"[All Fields]) OR ("depressive disorder, major"[MeSH Terms] OR ("depressive"[All Fields] AND "disorder"[All Fields] AND "major"[All Fields]) OR "major depressive disorder"[All Fields] OR ("Involutional"[All Fields] AND "psychosis"[All Fields]) OR "involutional psychosis"[All Fields]) OR ("depressive disorder, major"[MeSH Terms] OR ("depressive"[All Fields] AND "disorder"[All Fields] AND "major"[All Fields]) OR "major depressive disorder"[All Fields] OR ("psychoses"[All Fields] AND "Involutional"[All Fields]) OR "psychoses involutional"[All Fields]) OR ("depressive disorder, major"[MeSH Terms] OR ("depressive"[All Fields] AND "disorder"[All Fields] AND "major"[All Fields]) OR "major depressive disorder"[All Fields] OR ("depression"[All Fields] AND "Involutional"[All Fields]) OR "depression involutional"[All Fields]) OR ("depressive disorder, major"[MeSH Terms] OR ("depressive"[All Fields] AND "disorder"[All Fields] AND "major"[All Fields]) OR "major depressive disorder"[All Fields] OR ("Involutional"[All Fields] AND "depression"[All Fields]) OR "involutional depression"[All Fields]) OR ("depressive disorder, major"[MeSH Terms] OR ("depressive"[All Fields] AND "disorder"[All Fields] AND "major"[All Fields]) OR "major depressive disorder"[All Fields] OR ("melancholia"[All Fields] AND "Involutional"[All Fields])) OR ("depressive disorder, major"[MeSH Terms] OR ("depressive"[All Fields] AND "disorder"[All Fields] AND "major"[All Fields]) OR "major depressive disorder"[All Fields] OR ("Involutional"[All Fields] AND "melancholia"[All Fields]) OR "involutional melancholia"[All Fields]) | 83,401 |
| **#3** | **Dialectical Behavior Therapy:** "Dialectical Behavior Therapy"[MeSH Terms] OR ("Dialectical Behavior Therapy"[MeSH Terms] OR ("dialectical"[All Fields] AND "behavior"[All Fields] AND "therapy"[All Fields]) OR "Dialectical Behavior Therapy"[All Fields] OR ("behavior"[All Fields] AND "therapy"[All Fields] AND "dialectical"[All Fields]) OR "behavior therapy dialectical"[All Fields]) OR ("Dialectical Behavior Therapy"[MeSH Terms] OR ("dialectical"[All Fields] AND "behavior"[All Fields] AND "therapy"[All Fields]) OR "Dialectical Behavior Therapy"[All Fields] OR ("dialectical"[All Fields] AND "behavior"[All Fields] AND "therapies"[All Fields]) OR "dialectical behavior therapies"[All Fields]) | 1,124 |
| **#4** | **Psychotherapy:** "Psychotherapy"[MeSH Terms] OR "psychotherapie"[All Fields] OR "Psychotherapy"[MeSH Terms] OR "Psychotherapy"[All Fields] OR "psychotherapies"[All Fields] OR "psychotherapy s"[All Fields] | 274,928 |
| **#5** | **#1 AND #3 AND #4**: "depressive disorder, treatment resistant"[MeSH Terms] AND "Dialectical Behavior Therapy"[MeSH Terms] AND "Psychotherapy"[MeSH Terms] | 2 |
| **#6** | **#2 AND #3 AND #4**: "depressive disorder, major"[MeSH Terms] AND "Dialectical Behavior Therapy"[MeSH Terms] AND "Psychotherapy"[MeSH Terms] | 2 |
| **#7** | **#1 OR #2 AND #3 AND #4**: ("depressive disorder, treatment resistant"[MeSH Terms] OR "depressive disorder, major"[MeSH Terms]) AND "Dialectical Behavior Therapy"[MeSH Terms] AND "Psychotherapy"[MeSH Terms] | 3 |
| **#8** | **#1 AND #2 AND #3 AND #4**: "depressive disorder, treatment resistant"[MeSH Terms] AND "depressive disorder, major"[MeSH Terms] AND "Dialectical Behavior Therapy"[MeSH Terms] AND "Psychotherapy"[MeSH Terms] | 1 |
